# Volvulus du cæcum sur tumeur appendiculaire: à propos d'un cas rare

**DOI:** 10.11604/pamj.2014.19.199.4132

**Published:** 2014-10-24

**Authors:** Hicham El Bouhaddouti, Khalid El Haoudi

**Affiliations:** 1 Service de Chirurgie Viscérale, CHU Hassan II, Fès, Maroc

**Keywords:** Volvulus du cæcum, tumeur appendiculaire, syndrome occlusif, Cecal volvulus, appendiceal tumor, occlusive syndrome

## Image en medicine

Le volvulus du cæcum suite à une tumeur appendiculaire est une complication rare et une urgence chirurgicale dont l'origine est multifactorielle. Nous rapportons le cas d'un patient âgé de 59 ans, admis aux urgences pour un syndrome occlusif associé à des douleurs de la fosse iliaque droite. L'examen clinique avait objectivé une masse sensible de la fosse iliaque droite. Le scanner abdomino-pelvien avait révélé un aspect de volvulus caecal associé à une masse tissulaire latéro-caecale interne (A). Le patient a été opéré par laparotomie médiane avec découverte d'un volvulus du cæcum sur un processus appendiculaire (B), d'où la réalisation d'une hémi colectomie droite avec anastomose iléo colique termino-terminale. L'examen anatomo pathologique de la pièce opératoire est revenu en faveur d'un adénocarcinome mucineux de l'appendice, classé Pt3N1Mx. Le patient a bénéficié d'une chimiothérapie adjuvante, et après un recul de 12 mois, il est en bon contrôle clinico radiologique. L'intérêt de ce cas clinique est double, d'une part en raison de la rareté de cette pathologie et d'autre part dans la cause du volvulus du cæcum.

**Figure 1:**
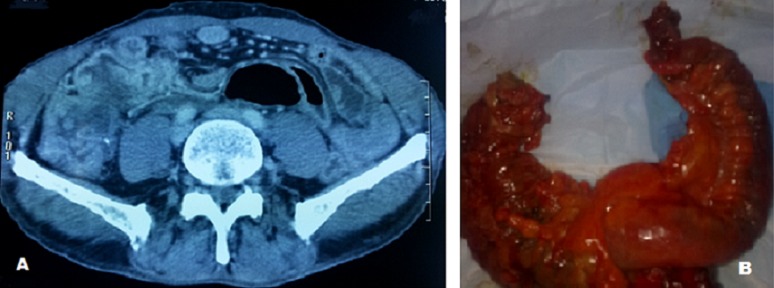
(A): aspect scannographique du volvulus du caecum sur un processus appendiculaire; (B): aspect per opératoire du volvulus du caecum

